# Sulforaphane Suppresses the Nicotine-Induced Expression of the Matrix Metalloproteinase-9 via Inhibiting ROS-Mediated AP-1 and NF-κB Signaling in Human Gastric Cancer Cells

**DOI:** 10.3390/ijms23095172

**Published:** 2022-05-05

**Authors:** Shinan Li, Pham Ngoc Khoi, Hong Yin, Dhiraj Kumar Sah, Nam-Ho Kim, Sen Lian, Young-Do Jung

**Affiliations:** 1 Research Institute of Medical Sciences, Chonnam National University Medical School, Gwangju 61469, Korea; 156103@chonnam.edu (S.L.); 197784@chonnam.edu (D.K.S.); nhk111@jnu.ac.kr (N.-H.K.); 2 Faculty of Basic Medical Sciences, Pham Ngoc Thach University of Medicine, Ho Chi Minh City 740500, Vietnam; khoicnsh@gmail.com; 3 Department of Biochemistry and Molecular Biology, School of Basic Medical Sciences, Southern Medical University, Guangzhou 510515, China; graceyinh@126.com; 4 Department of Biochemistry, Chonnam National University Medical School, Hwasun 58128, Korea

**Keywords:** sulforaphane, nicotine, metalloproteinase-9, gastric cancer, cell invasion

## Abstract

Sulforaphane, a natural phytochemical compound found in various cruciferous vegetables, has been discovered to present anti-cancer properties. Matrix metalloproteinase-9 (MMP-9) plays a crucial role in gastric cancer metastasis. However, the role of sulforaphane in MMP-9 expression in gastric cancer is not yet defined. Nicotine, a psychoactive alkaloid found in tobacco, is associated with the development of gastric cancer. Here, we found that sulforaphane suppresses the nicotine-mediated induction of MMP-9 in human gastric cancer cells. We discovered that reactive oxygen species (ROS) and MAPKs (p38 MAPK, Erk1/2) are involved in nicotine-induced MMP-9 expression. AP-1 and NF-κB are the critical transcription factors in MMP-9 expression. ROS/MAPK (p38 MAPK, Erk1/2) and ROS functioned as upstream signaling of AP-1 and NF-κB, respectively. Sulforaphane suppresses the nicotine-induced MMP-9 by inhibiting ROS-mediated MAPK (p38 MAPK, Erk1/2)/AP-1 and ROS-mediated NF-κB signaling axes, which in turn inhibit cell invasion in human gastric cancer AGS cells. Therefore, the current study provides valuable evidence for developing sulforaphane as a new anti-invasion strategy for human gastric cancer therapy.

## 1. Introduction

Gastric cancer is the most common gastrointestinal cancer with high mortality worldwide. The vast majority of gastric cancer cases detected are in the advanced stage [1]. The poor prognosis and treatment of gastric cancer are highly relative to metastasis [2]. Therefore, exact studies on the mechanisms underlying gastric cancer may contribute to the development of improved treatments. Tobacco abuse is strongly associated with gastric cancer progression [3]. Nicotine, a psychoactive alkaloid contained in tobacco, is a known carcinogen, related to cancer metastasis, including gastric cancer [4]. It has been reported that the pathophysiological roles of nicotine are mediated by nicotinic acetylcholine receptors [5]. In addition, nicotine promotes invasion and angiogenesis by the induction of cyclooxygenase-2 and vascular endothelial growth factor receptor-2 in gastric cancer [6].

Tumor metastasis includes the cancer progression of proliferation, migration, invasion, and the following progression of adhesion and angiogenesis in a distant tissue [2]. Most cancer-related mortalities are due to tumor metastasis. Studies indicate that cell invasion is one of the fundamental properties of malignant cancer cells [7]. The breakdown of the extracellular matrix by proteinases is an essential step in cancer cell invasion [8]. Matrix metalloproteinases (MMPs), a family of extracellular-matrix-degrading proteinases, induce cancer cell invasion through the degradation of the extracellular matrix and the basal membrane [9]. Among the MMPs, MMP-2, and MMP-9 play crucial roles in cancer metastasis [10]. Normally, MMP-2 is constitutively expressed in highly malignant tumors, whereas MMP-9 is induced by growth factors [11]. High MMP-9 expression is observed in tumor extracts in gastric cancer; furthermore, aberrant expression of MMP-9 can increase tumor cell detachment and metastasis, which are related to malignancy and poor prognosis in gastric cancer [12]. Therefore, agents with the ability to suppress MMP-9 expression may be useful for the development of treatment strategies for gastric cancer.

Sulforaphane, a natural compound that is abundant in cruciferous vegetables, exhibits several beneficial properties, including anti-inflammatory, antioxidant, and anticancer activities. Recently, the anticancer effect of sulforaphane has attracted much attention [13]. Intake of cruciferous vegetables has been proven to prevent gastric cancer. It has been reported that sulforaphane induces cell cycle arrest and apoptosis in human colorectal cancer cells [14]. Sulforaphane was also suggested to sensitize hepatoma cancer cells to TRAIL-mediated apoptosis by reactive oxygen species (ROS)-mediatedDR5 expression [15]. Our recent studies show that sulforaphane inhibits bladder cancer cell proliferation via suppression of HIF-1α-mediated glycolysis in hypoxia [16]. Moreover, sulforaphane shows a protective effect on gastric mucosa via the Nrf2 mechanism [17]. The effects of MMP-9 inhibitors on the treatment of gastric cancer have been widely reported. However, the potential mechanisms by which sulforaphane inhibits MMP-9 expression are not fully understood in gastric cancer.

In this study, we examined the effect of sulforaphane on nicotine-mediated induction of MMP-9 and explored the underlying mechanisms. Based on our results, we reported that sulforaphane suppresses the nicotine-induced MMP-9 by inhibiting ROS-mediated MAPK (p38 MAPK, Erk1/2)/AP-1 and ROS-mediated NF-κB signaling axes, which in turn inhibit cell invasion in human gastric cancer AGS cells.

## 2. Results

### 2.1. Sulforaphane Suppresses Nicotine-Induced MMP-9 Expression in AGS Cells

**Figure 1:**
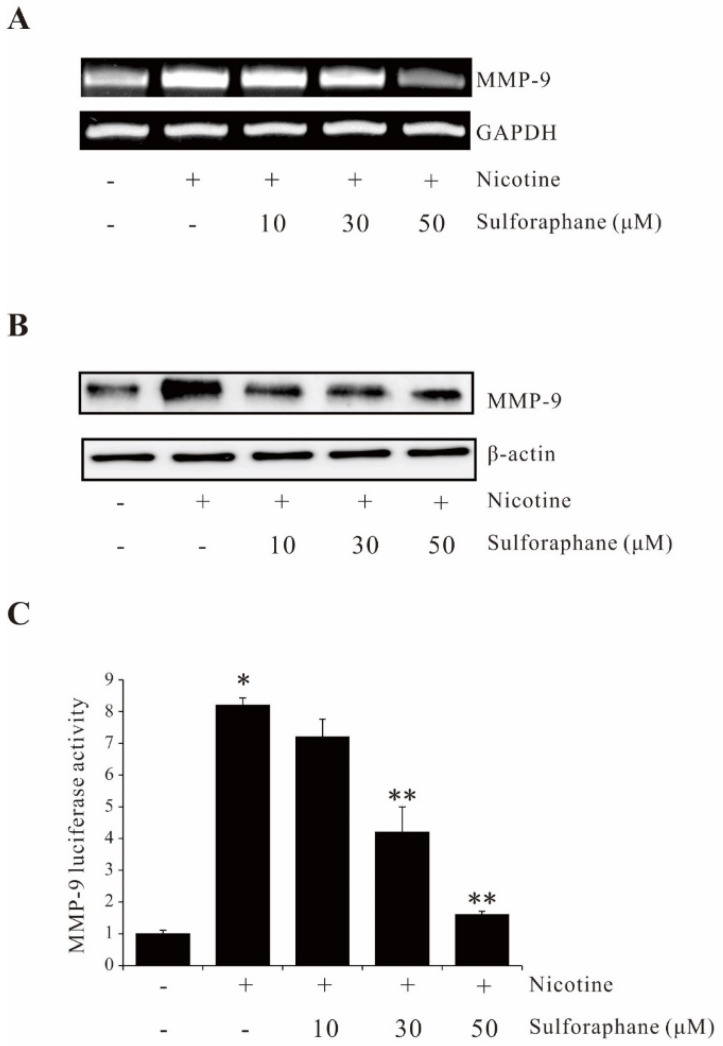
Sulforaphane inhibits nicotine-induced MMP-9 expression in human gastric AGS cells. AGS cells were pretreated with sulforaphane (10, 30, 50 µM) for 1 h, followed by treatment with 100 µg/mL nicotine for 4 h or 12 h, and MMP-9 expression was analyzed by performing RT-PCR (**A**), Western blot (**B**), and luciferase activity assay (**C**), respectively. The data represent the mean ± SEM from three experimental trials. * *p* < 0.05 in comparison with the control; ** *p* < 0.05 in comparison with nicotine alone.

To determine the effect of sulforaphane on nicotine-induced MMP-9 expression, AGS cells were pretreated with sulforaphane and treated with nicotine. The expression levels of MMP-9 mRNA and protein were measured, respectively. As shown in Figure 1A,B, treatment with nicotine significantly induced MMP-9 expression at both the mRNA and protein levels, which can be abrogated by sulforaphane. In addition, suppression of nicotine-induced MMP-9 promoter activity was also observed in sulforaphane-pretreated AGS cells (Figure 1C). These results showed that sulforaphane inhibits nicotine-induced MMP-9 expression in human gastric cancer AGS cells.

### 2.2. Sulforaphane Suppresses Nicotine-Induced MMP-9 Expression by Inhibiting ROS Generation

**Figure 2:**
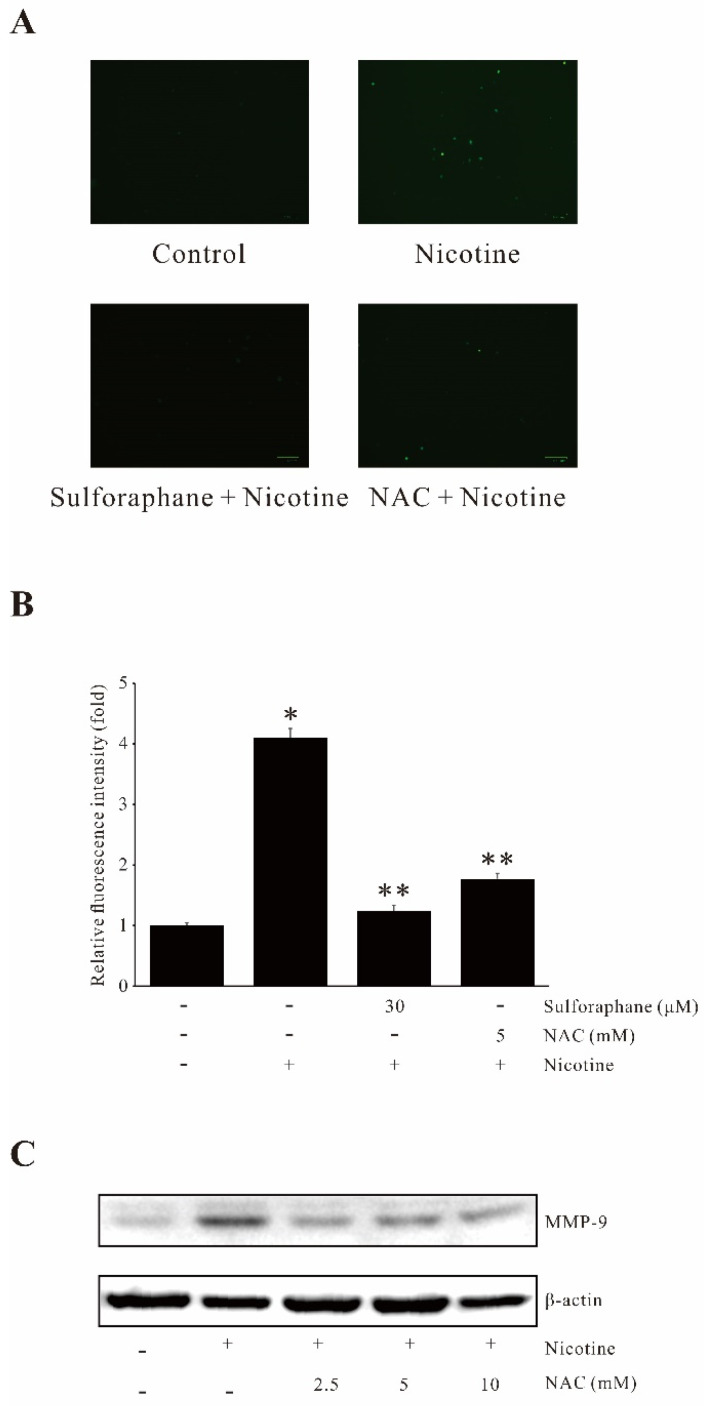
Sulforaphane inhibits nicotine-induced ROS in human gastric cancer AGS cells. (**A**) AGS cells were pretreated with 30 µM sulforaphane and 5 mM NAC for 1 h prior to nicotine treatment for 30 min. The cells were then incubated in the dark for 10 min with 10 µM H_2_DCFDA. The H_2_DCFDA fluorescence was imaged using a confocal laser scanning fluorescence microscope. (**B**) Relative fluorescence intensities of the ROS production level. (**C**) Cells pretreated with NAC (2.5, 5, 10 mM) for 1 h were incubated with nicotine (100 µg/mL) for 12 h. After incubation, extracted proteins were analyzed for the induction of MMP-9 expression by Western blot. The data represent the mean ± SEM from three experimental trials. * *p* < 0.05 in comparison with the control; ** *p* < 0.05 in comparison with nicotine alone.

Due to the important role of oxidative stress in the pathogenesis of cancer [18], the ROS production levels were determined by H_2_DCFDA treated with nicotine in the presence or absence of sulforaphane. As shown in Figure 2A,B, sulforaphane suppressed the nicotine-induced ROS production levels. N-Acetylcysteine (NAC) was used as a positive control. NAC treatment abrogated nicotine-induced MMP-9 expression (Figure 2C). These results indicate that sulforaphane suppressed the nicotine-induced MMP-9 via regulating ROS generation in human gastric cancer AGS cells. 

### 2.3. Sulforaphane Suppresses Nicotine-Induced MMP-9 Expression by Inhibiting p38 MAPK and Erk1/2 Activation

**Figure 3:**
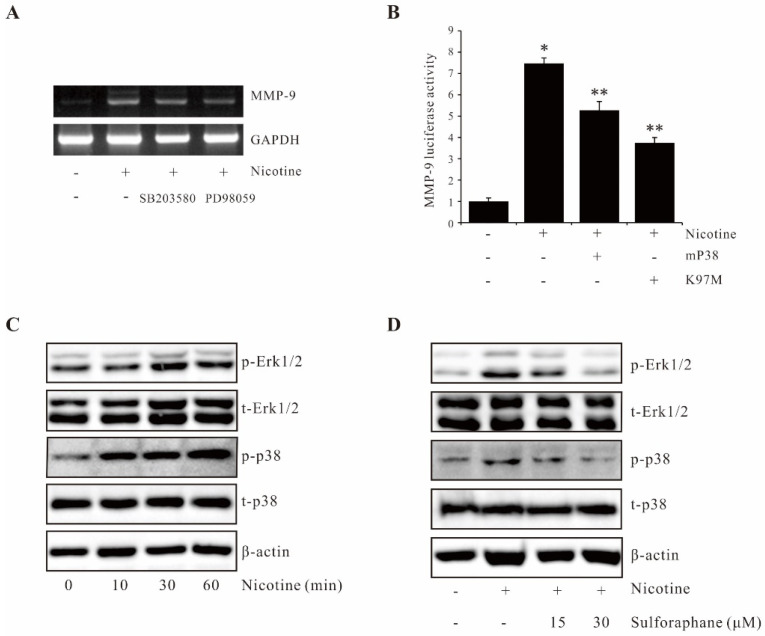
Sulforaphane inhibits nicotine-induced MMP-9 expression by suppressing p38 MAPK and Erk1/2 signaling pathways. (**A**) AGS cells were pretreated with 20 µM SB203580 and 20 µM PD 98059 for 1 h and incubated with 100 µg/mL nicotine for 4 h. After incubation, extracted mRNA was analyzed for the induction of MMP-9 expression by RT-PCR. (**B**) AGS cells were cotransfected with dominant-negative mutants of p38 MAPK (mP38) or MEK-1 (K97M) and the pGL4-MMP-9 promoter-reporter construct. The luciferase activity was determined using a luminometer after incubating the cells with 100 µg/mL nicotine for 4 h. (**C**) Cells were treated with 100 µg/mL nicotine for 0–60 min, and extracted proteins were analyzed by Western blot. (**D**) Cells were pretreated with sulforaphane (15, 30 µM) followed by 100 µg/mL nicotine treatment for 30 min, and extracted proteins were analyzed by Western blot. The data represent the mean ± SEM from three experimental trials. * *p* < 0.05 in comparison with the control; ** *p* < 0.05 in comparison with nicotine alone.

MAPKs have well-established roles in the progression of human cancers [19,20]. To determine the role of MAPKs on nicotine-induced MMP-9 expression, pharmacological inhibitors of MAPKs, SB203580 (a p38 MAPK inhibitor), and PD98059 (a MEK inhibitor) were used along with nicotine treatment in AGS cells. As shown in Figure 3A, both SB203580 and PD98059 inhibited the nicotine-induced MMP-9 expression at the transcriptional level. Transfection of dominant-negative mutant constructs mP38 (p38 MAPK) or K97M (MEK-1) attenuated nicotine-induced MMP-9 promoter activity (Figure 3B). Additionally, we found that sulforaphane suppressed nicotine-induced p38 MAPK and Erk1/2 (Figure 3C,D). These results suggest that sulforaphane suppressed nicotine-induced MMP-9 expression by inhibiting p38 MAPK and Erk1/2 activation in AGS cells.

### 2.4. Sulforaphane Suppresses Nicotine-Induced MMP-9 Expression by Inhibiting Reporter Activities of AP-1 and NF-κB

**Figure 4:**
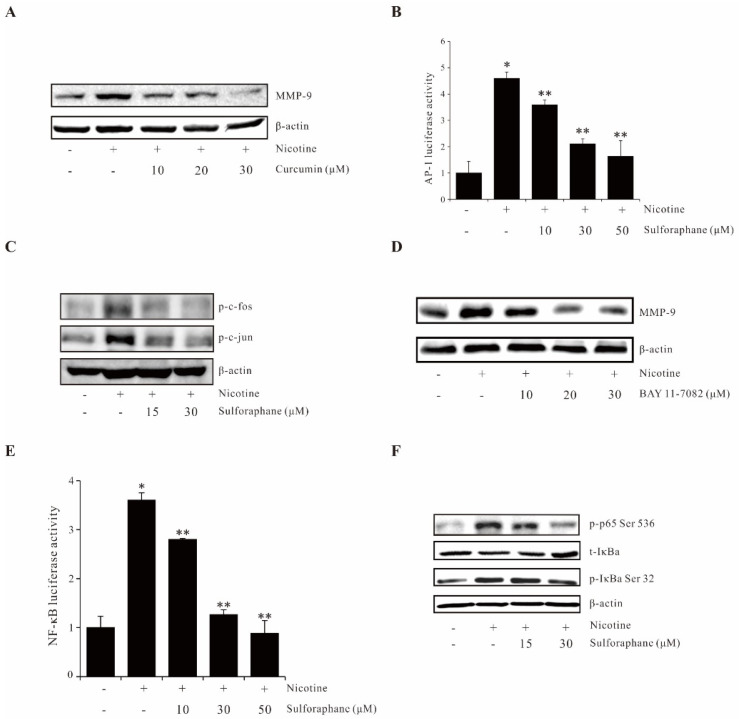
Sulforaphane inhibits nicotine-induced MMP-9 expression by suppressing the transcription factors of AP-1 and NF-κB. (**A**) AGS cells were pretreated with the indicated concentration of curcumin and treated with 100 µg/mL nicotine for 12 h, and extracted proteins were analyzed for the induction of MMP-9 expression by Western blot. (**B**) Cells were transfected with the AP-1 luciferase reporter. The luciferase activity was determined using a luminometer after incubating the cells with sulforaphane for 1 h prior to nicotine treatment for 4 h. (**C**) Cells were pretreated with sulforaphane and treated with 100 µg/mL nicotine for 1 h; the expression of phos-c-fos and phos-c-jun were analyzed by Western blot. (**D**) Cells were pretreated with the indicated concentration of BAY11-7082 and treated with 100 µg/mL nicotine for 12 h, and extracted proteins were analyzed for the induction of MMP-9 expression by Western blot. (**E**) Cells were transfected with the NF-κB luciferase reporter. The luciferase activity was determined using a luminometer after incubating the cells with sulforaphane for 1 h prior to nicotine treatment for 4 h. (**F**) Cells were pretreated with sulforaphane and treated with 100 µg/mL nicotine for 1 h, the expression of phos-p65 (Ser536 and phos-IκBα (Ser32) were analyzed by Western blot. The data represent the mean ± SEM from three experimental trials. * *p* < 0.05 in comparison with the control; ** *p* < 0.05 in comparison with nicotine alone.

Studies showed that AP-1 plays a pivotal role in tumor carcinogenesis [21]. Curcumin (an AP-1 inhibitor) pretreatment significantly suppressed the nicotine-induction MMP-9 protein expression and promoter activity (Figure 4A,B). Furthermore, sulforaphane treatment resulted in significant inhibition of nicotine-induced c-fos and c-jun phosphorylation. (Figure 4C). Moreover, NF-κB is also a key transcription factor in tumor carcinogenesis [22]. BAY 11-7082 (an NF-κB inhibitor) pretreatment decreased the nicotine-induced MMP-9 protein expression and promoter activity (Figure 4D,E). It is observed that sulforaphane suppressed the nicotine-enhanced phosphorylation of NF-κB and IκBα (Figure 4F). These results demonstrated that sulforaphane inhibited nicotine-induced MMP-9 expression via suppressing AP-1 and NF-κB activation.

### 2.5. ROS/(p38 MAPK, Erk1/2) and ROS Functioned as Upstream Regulators of AP-1 and NF-κB Respectively

**Figure 5:**
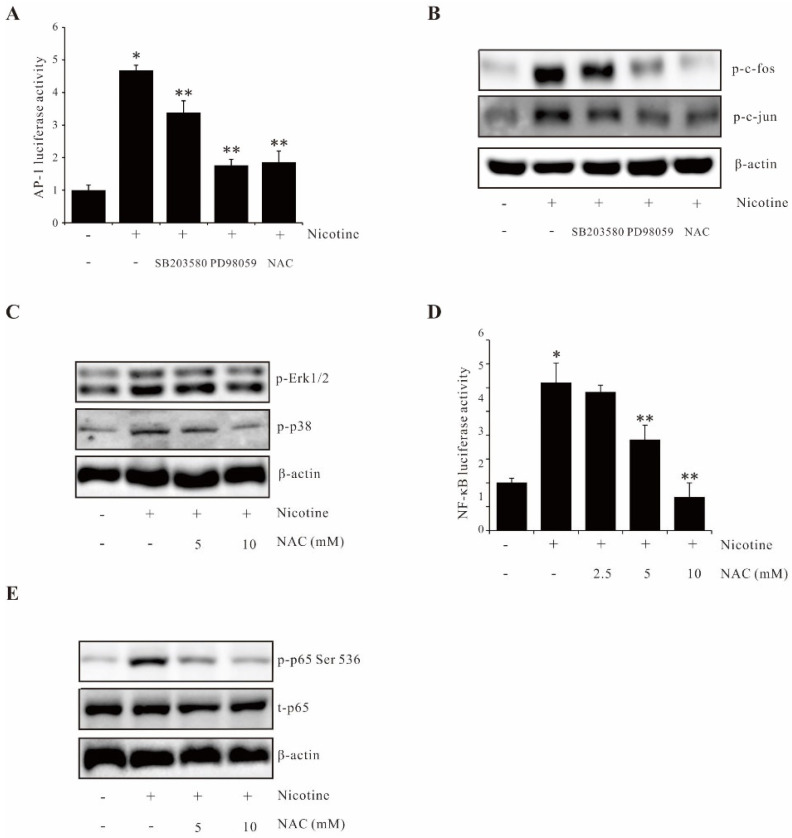
ROS/(p38 MAPK, Erk1/2) and ROS functioned as upstream regulators of AP-1 and NF-κB, respectively. (**A**) AGS cells were transfected with the AP-1 luciferase reporter. The luciferase activity was determined using a luminometer after incubating the cells with 20 µM SB203580, 20 µM PD 98059, or 5 mM NAC for 1 h prior to nicotine treatment for 4 h. (**B**) AGS cells were pretreated with 20 µM SB203580, 20 µM PD 98059, or 5 mM NAC and treated with 100 µg/mL nicotine for 30 min; the expression of phos-c-fos and phos-c-jun were analyzed by Western blot. (**C**) Cells were pretreated with the indicated concentration of NAC and treated with 100 µg/mL nicotine for 30 min; the expression of phos-Erk1/2 and phos-p38 were analyzed by Western blot. (**D**) Cells were transfected with the NF-κB luciferase reporter. The luciferase activity was determined using a luminometer after incubating the cells with NAC for 1 h prior to nicotine treatment for 4 h. (**E**) Cells were pretreated with the indicated concentration of NAC and treated with 100 µg/mL nicotine for 30 min; the expression of phos-p65 (Ser5360 and phos-IκBα (Ser32) were analyzed by Western blot. The data represent the mean ± SEM from three experimental trials. * *p* < 0.05 in comparison with the control; ** *p* < 0.05 in comparison with nicotine alone.

To dissect the relevant signaling pathways contributing to AP-1 activation induced by nicotine, we performed inhibitor studies with luciferase activity assay and Western blot. As shown in Figure 5A, SB203580 partially suppressed the AP-1 transcription, while both PD98059 and NAC significantly blocked the AP-1 transcription. Similar results are shown at protein levels (Figure 5B). In addition, the data presented in Figure 5C indicated that the ROS inhibitor, NAC, decreases p38 MAPK and Erk1/2 activation. These results indicate that ROS/(p38 MAPK, Erk1/2) is the upstream regulator of AP-1 in nicotine-induced MMP-9 expression in AGS cells. Next, we examined which relevant regulator contributed to AP-1 activation induced by nicotine. To determine whether the ROS contributed to NF-κB activation induced by nicotine, the effects of an inhibitor of ROS on nicotine-induced NF-κB activation were examined. The inhibitor of ROS, NAC, inhibited nicotine-mediated NF-κB reporter activity (Figure 5D). Pretreatment of NAC attenuated nicotine-media activation of p65 (Figure 5E). These findings supported that ROS functioned as upstream signaling of NF-κB in nicotine-induced MMP-9 expression in AGS cells.

### 2.6. Sulforaphane Attenuates the Invasiveness of AGS Cells by Suppressing MMP-9 Expression

**Figure 6:**
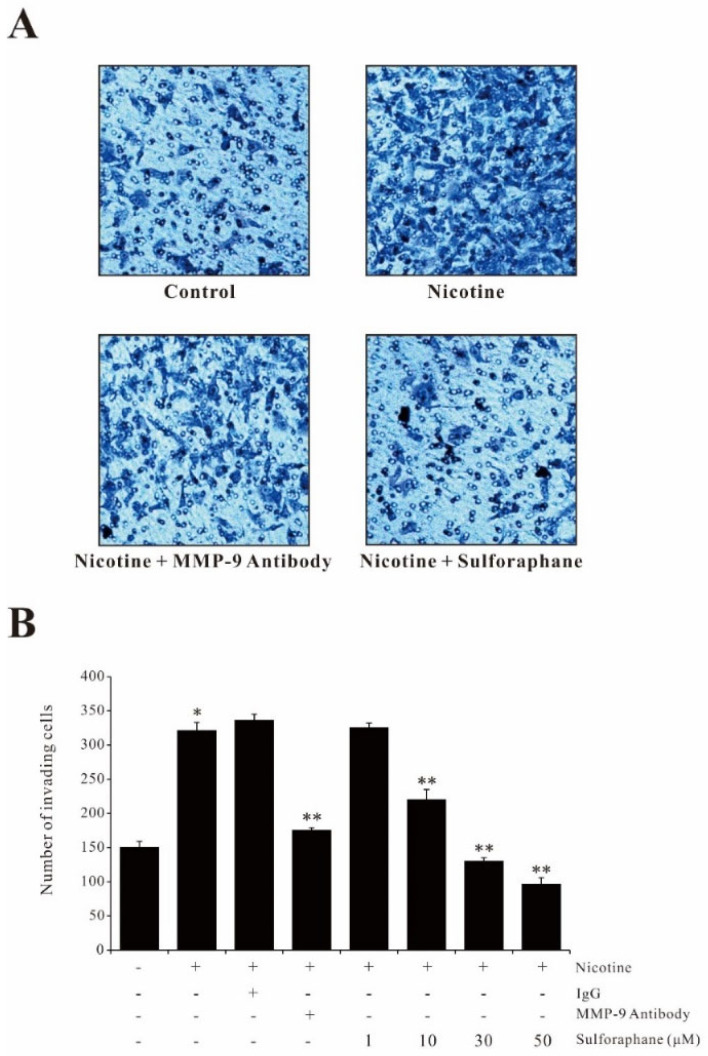
Sulforaphane inhibits nicotine-induced cell invasion in AGS cells. (**A**) AGS cells (3 × 10^5^) were incubated with nicotine (100 µg/mL) in the presence or absence of sulforaphane (30 µM) or anti-MMP-9 antibody (200 ng/mL) in a Corning Matrigel matrix for 24 h. (**B**) AGS cells (3 × 10^5^) were incubated with nicotine (100 µg/mL) in the presence or absence of nonspecific IgG, anti-MMP-9 antibody (200 ng/mL), or sulforaphane (10, 30, 50 µM) in a Corning Matrigel matrix for 24 h. Cells invading the undersurface of the membrane were stained using a Diff-Quick stain kit and counted under a phase-contrast light microscope. The data represent the mean ± SEM from three experimental trials. * *p* < 0.05 in comparison with the control; ** *p* < 0.05 in comparison with nicotine alone.

It is well known that high expression of MMP-9 is important for the invasive phenotype of cancer cells. The effect of sulforaphane on nicotine-induced cell invasion was examined by performing a matrigel invasion assay. AGS cells incubated in nicotine resulted in increased activity of the cell invasive phenotype. However, in the presence of sulforaphane or an MMP-9 antibody, the number of invaded cells decreased, suggesting that sulforaphane suppressed the nicotine-induced invasive phenotype by inhibiting MMP-9 expression (Figure 6A). We further counted the invading cells and the cell invasion results showed with statistically significant values that the sulforaphane pretreatment significantly reduced the nicotine-induced cell invasive activity as well as the neutralizer, MMP-9 antibody (Figure 6B). These results further indicated that sulforaphane inhibited the AGS cell invasive activity by downregulating the MMP-9 expression.

## 3. Discussion

Gastric cancer ranks as the fourth most common cancer and is one of the leading causes of cancer-related death worldwide [23]. Phytochemicals, derived from plants, have become an important source of anticancer medicines, with antioxidant activities [24]. Sulforaphane, 1-isothiocyanato-4-(methylsulfinyl)butane, a natural compound that includes the isothiocyanate group of organosulfur compounds, is one of the major phytochemicals found in cruciferous vegetables [25]. Many studies have been directed at defining the role of sulforaphane as an anticancer medicine in humans, due to various reasons. Firstly, cruciferous vegetables, particularly broccoli, are rich in sulforaphane, which can prevent cancer risk [26]. Sulforaphane may protect against various types of cancer. In breast cancer, combination therapy with sulforaphane has been shown to improve the outcome [27]. Sulforaphane can inhibit breast cancer stem cells via downregulation of the Wnt/β-catenin self-renewal pathway in the xenograft mice model [28]. In colorectal cancer, sulforaphane inhibits the stemness of cancer stem cells both in vitro and in vivo by targeting TAp63α [29]. Rutz et al. reported that sulforaphane acts as a histone deacetylase (HDAC) inhibitor to prostate cancer cell progression [30]. In addition, sulforaphane has a potential therapeutic application in the treatment and prevention of gastric cancer by induction of apoptosis of gastric cancer cells [31]. Our earlier studies indicated that sulforaphane decreased glycolytic metabolism in a hypoxia microenvironment by inhibiting hypoxia-induced HIF-1α and HIF-1α trans-localization in non-muscle-invasive bladder cancer cell lines [16]. Moreover, it has been known that sulforaphane has many health benefits. Sulforaphane could prevent memory dysfunction and improve cognitive function [32]. Sulforaphane prevents type 2 diabetes-induced cardiomyopathy by activating the lipid metabolic pathway and enhancing NRF2 activation [33]. Sulforaphane presents anti-inflammation properties by suppression of cyclooxygenase-2 expression [34]. 

Clinical and epidemiological research has revealed that smokers are more likely to develop cancer progression as compared to non-smokers [35]. Cigarette smoke caused many diseases and cancers, and nicotine is a major poison in cigarette smoke [36]. Nicotine caused more harm to human organs and tissues than other compounds of cigarette smoke [37]. Recently, we demonstrated that nicotine promotes gastrointestinal cancer progression through IL-8 upregulation [38]. Aberrant processes of wound healing contribute to cancer progression [39]. Matrix metalloproteinases (MMPs) have been identified as the main factors in both acute and chronic wounds and the excess protease activity can lead to a chronic nonhealing wound [40]. Reiss et al. reported that when MMP-9 is expressed at excessive levels, it prevents the reestablishment of the dermal/epidermal junction and, thereby, limits epithelial migration and wound closure in a murine wound model [41].

In the present study, we attempted to explore the role and potential mechanisms of sulforaphane in nicotine-challenged gastric cancer cells. We revealed that nicotine induces MMP-9 expression and cell invasiveness in gastric cancer AGS cells. Sulforaphane effectively suppressed ROS, p38 MAPK, Erk1/2, AP-1, and NF-κB activation by inhibiting MMP-9 expression in gastric cancer AGS cells.

Healthy bodies and tissues are often subjected to sublethal doses of various oxidants [42]. There is considerable evidence suggesting oxidative stress has been associated with the development of cancer [18]. Increased ROS generation was observed in cancer cells compared with normal cells [43]. ROS function as secondary messengers and control various signaling cascades in cells [44]. Nicotine promotes atherosclerosis by the induction of ROS in endothelial cells [45]. The present study suggested that nicotine induces ROS generation in gastric cancer AGS cells, and NAC abrogated nicotine-induced MMP-9 expression. Sulforaphane suppresses ROS production to inhibit nicotine-induced MMP-9 expression. NADPH oxidases were identified as upstream signal molecules of ROS in AGS cells. NADPH oxidase activation is regulated by several processes such as phosphorylation of its components, exchange of GDP/GTP on Rac2, and binding of p47^phox^ and p40^phox^ to phospholipids [46]. Membrane translocation of p47^phox^ plays a critical role in the activation of NADPH oxidase [47]. Nicotine can trigger the generation of ROS through NADPH oxidase [48]. Sulforaphane decreases ROS and inhibits carcinogenesis by the activation of NRF2 [49]. Sulforaphane was also reported to induce HO-1 in microglia [50]. In T24 bladder cancer cells, sulforaphane upregulates ROS to induce cell apoptosis [51]. Sulforaphane induces ROS generation to promote tumor necrosis factor-related apoptosis-inducing ligand sensitivity [52]. In this respect, the mechanisms involved in sulforaphane inhibited nicotine-activated ROS are revealed in this study.

MAPK cascade plays a vital role in various cancer progression [20]. MAPK-regulated MMP-9 in cancer cells has been reported in many studies [7]. Here, nicotine stimulated the phosphorylation of p38 MAPK and Erk1/2 to induce MMP-9 expression in AGS cells. The aberrant activation of EGFR has been implicated in tumor growth [53]. Previously, we observed that EGFR is involved in MMP-9 expression in human endothelial cells [54]. One study reported that Akt and PKCδ are associated with TPA-induced MMP-9 expression [55]. MAPKs are studied as the downstream of PKCα/β [56,57]. Experiments on colorectal tumor cells documented that MAPK signaling may directly depend on ROS [58]. Treatment with sulforaphane significantly reduced the amount of phosphorylated Akt and phosphorylation of the mTOR subunit [59]. In this study, sulforaphane inhibited p38 MAPK and Erk1/2 activation to suppress nicotine-induced MMP-9 expression. Thus, many additional signaling modulators should be explored to define sulforaphane suppression of nicotine-induced MMP-9 expression in AGS gastric cancer cells.

Our previous study revealed the important role of AP-1 and NF-κB in regulating MMP-9 by cadmium in endothelial cells [54]. AP-1 is composed of members of the c-fos and c-jun families, which have been shown to regulate the expression of several genes involved in tumor development. Here, enhanced phosphorylation of c-fos and c-jun was observed in nicotine-treated cells. Our results showing that AP-1 inhibitor ameliorated MMP-9 expression indicated that AP-1 contributed to nicotine-mediated induction of MMP-9. Sulforaphane’s inhibition of c-fos and c-jun phosphorylation accompanied by a reduction in AP-1 transcription factor activity, therefore, suppressed MMP-9 expression. To further determine the underlying mechanisms, we treated AGS cells with the inhibitors of ROS, p38 MAPK, and Erk1/2. We found that inhibitors of ROS, p38 MAPK, and Erk1/2 suppressed the nicotine-mediated c-fos and c-jun activation and the AP-1 reporter activity. ROS inhibitor reduced the nicotine-induced activation of p38 MAPK and Erk1/2. Our results indicated that ROS/MAPK (p38 MAPK, Erk1/2) functioned as the upstream signaling molecules in the nicotine-activated AP-1 pathway. Src tyrosine was reported to be upstream of AP-1 [60]. We observed that JNK1/2 mediates AP-1 activation in AGS cells [61]. Increased NF-κB translocation is usually associated with its phosphorylation and IκB proteasomal degradation in many types of cancer progression. In our study, we found that IκBα and p65, the two subunit elements of NF-κB, play vital roles in the nicotine-mediated induction of MMP-9 in AGS cells. It was observed that sulforaphane inhibited the nicotine-induced NF-κB p65 and IκBα in a dose-dependent manner. ROS contribute to the upstream signaling of NF-κB [62]. Our results showed that ROS are the upstream molecules of NF-κB in nicotine-induced MMP-9 in AGS cells. A prior study suggested that the EGFR signaling activates NF-κB via mTORC2 [63]. We found that Erk1/2 and JNK were critical for NF-κB in bladder cancer cells [64].

## 4. Conclusion

**Figure 7:**
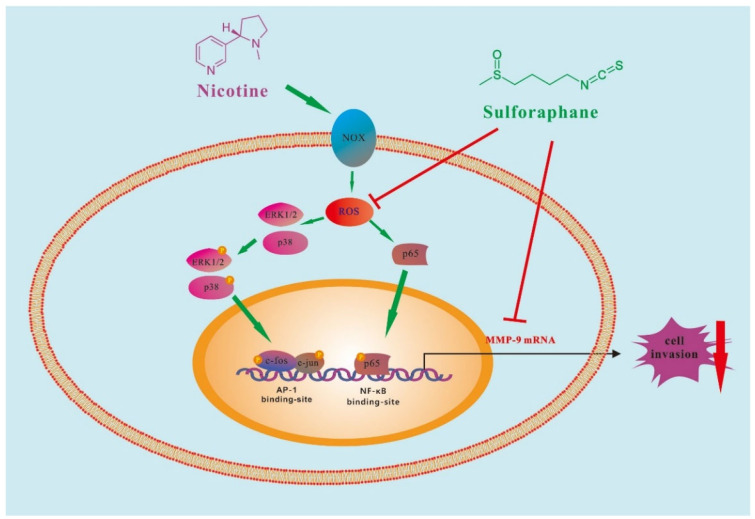
Schematic representation of the mechanism underlying the inhibition of nicotine-induced MMP-9 expression by sulforaphane in AGS cells. Sulforaphane inhibits nicotine-induced MMP-9 expression via suppression of the ROS/MAPKs(p38 MAPK, Erk1/2)/AP-1 and ROS/NF-κB signaling pathways, which in turn attenuate AGS cell invasiveness.

Figure 7 illustrates that sulforaphane suppresses the nicotine-induced MMP-9 by inhibiting ROS-mediated MAPK (Erk1/2, p38 MAPK)/AP-1 and ROS-mediated NF-κB signaling axes, which in turn inhibit cell invasion in human gastric cancer AGS cells. These findings demonstrate that sulforaphane might be a potential functional food ingredient with the feature of gastric cancer therapy.

## 5. Materials and Methods

### 5.1. Reagents

RPMI-1640, OPTI-modified Eagle’s medium, fetal bovine serum (FBS), phosphate-buffered saline, and penicillin–streptomycin solution were obtained from HyClone (Logan, UT, USA). TrypLE™ Express was obtained from Gibco (Grand Island, NY, USA). Sulforaphane, nicotine, DMSO, curcumin, and all other chemicals were purchased from Sigma-Aldrich (St. Louis, MO, USA). BAY11-7082, PD98059, and SB203580 were purchased from Calbiochem (San Diego, CA, USA). Antibodies against MMP-9, phos-Erk1/2, Erk1/2, phos-p38, p38, phos-c-jun, phos-c-fos, phos-p65 (Ser536), phos-IκBα (Ser32), and IκBα were purchased from Cell Signaling Technology (Danvers, MA, USA).

### 5.2. Cell Culture

The AGS human gastric cancer cell line was obtained from American Type Culture Collection (Manassas, VA, USA) and cultured in RPMI-1640 medium supplemented with 10% fetal bovine serum (FBS) and 0.6% penicillin–streptomycin at 37 °C in a 5% CO_2_ humidified incubator. In these experiments, stimulants such as nicotine were added to serum-free media for the indicated time intervals. When the inhibitors were used, they were added 1 h before the nicotine treatment.

### 5.3. Reverse Transcription PCR

AGS cells were treated with 100 µg/mL nicotine for 4 h. Then, total RNA was extracted from the AGS cells using TRIzol reagent (Invitrogen). One µg of total RNA was used for first-strand complementary DNA synthesis using random primers and M-MLV transcriptase (Promega). The complementary DNA was subjected to PCR amplification with primer sets for GAPDH and MMP-9 using a PCR master mix solution (iNtRON, Korea). The specific primer sequences were as follows: GAPDH sense, 5′-TTG TTG CCA TCA ATG ACC CC-3′, and GAPDH antisense, 5′-TGA CAA AGT GGT CGT TGA GG-3′ (836 bp); and MMP-9 sense, 5′- AAG TGG CAC CAC CAC AAC AT -3′ and MMP-9 anti-sense, 5′-TTT CCC ATC AGC ATT GCC GT-3′ (497 bp). The PCR conditions were as follows: denaturation at 94 °C for 30 s, annealing at 52 °C for 20 s, and extension at 72 °C for 30 s, 28 cycles.

### 5.4. Western Blot Analysis

AGS cells were treated with 100 µg/mL nicotine for 12 h to detect the MMP-9 changes and were treated with 100 µg/mL nicotine for 30–60 min to detect the signal molecule changes. After each experiment, cells were washed twice with cold PBS and were harvested in 100 µL of protein extraction solution (iNtRON, Seongnam, Korea). Cell homogenates were centrifuged at 10,000× *g* for 20 min at 4 °C. Equal amounts of total cellular protein (50 µg) were electrophoresed in sodium dodecyl sulfate (SDS)-polyacrylamide gels, and the protein was then transferred to polyvinylidene difluoride membranes (Millipore, Billerica, MA, USA). Nonspecific binding sites on the membranes were blocked with 5% nonfat dry milk in 15 mM Tris/150 mM NaCl buffer (pH 7.4) at room temperature for 2 h. Membranes were incubated with the target antibody. The membranes were then probed with a secondary antibody labeled with horseradish peroxidase. The bands were visualized using an enhanced chemiluminescence kit (Millipore, Billerica, MA, USA) and were scanned by a luminescence image analyzer (Vilber Lourmat, Collégien, France). 

### 5.5. Transient Transfection with Dominant Negative Mutants

The plasmids encoding dominant-negative mutants of MEK-1 (pMCL-K97M) and p38 MAPK (pMCL-mP38) were kindly provided by Dr. N. G. Ahn (University of Colorado, Boulder, CO, USA) and Dr. J. Han (Scripps Research Institute, CA, USA), respectively. All mutants were prepared by using TIANGEN (Beijing, China) plasmid DNA preparation kits. Dominant-negative mutants (1 µg) were carried out using Lipofectamine 3000 from Invitrogen (Carlsbad, CA, USA).

### 5.6. Measurement of MMP-9, AP-1 and NF-κB Luciferase Activity

The transcriptional regulation of MMP-9 was examined by the transient transfection of an MMP-9 promoter–luciferase reporter construct (pGL4-MMP-9). The plasmid pGL4-MMP-9 promoter (spanning nucleotides from −925 to +13) was kindly provided by Dr. Young-Han Lee (Konkuk University, Korea). The NF-κB and AP-1 luciferase reporter plasmid were purchased from Clontech (Palo Alto, CA, USA). The effects of sulforaphane on MMP-9 promoter activity were determined by pretreating cells with sulforaphane for 1 h prior to the nicotine treatment. Cells were collected with cell culture lysis reagent (Promega, Madison, WI, USA) and the luciferase activity was determined using a luminometer (Centro XS lb960 microplate luminometer, Berthold Technologies, Oak Ridge, TN, USA) according to the manufacturer’s protocol.

### 5.7. Detection of ROS by H_2_DCFDA

ROS production levels were performed by modifying the method described by our previous study [65]. Briefly, H_2_DCFDA (MCE, Romulu, MI, USA), a cell-permeable probe, was used to detect changes in intracellular ROS produced by AGC cells in the sulforaphane and nicotine treatment group (30 min) and control, which were incubated with H_2_DCFDA at 37 °C with 5% CO_2_ for 30 min, digested with trypsin, and suspended in PBS. Images were acquired using the Laser Scanning Microscope 5 PASCAL program (Carl Zeiss) and a confocal microscope. DCF fluorescence was excited at 488 nm with an argon laser, and the evoked emission was filtered with a 515 nm long-pass filter.

### 5.8. Matrigel Invasion Assay

The cell invasion assay was carried out according to our previous study [66] using 10-well chemotaxis chambers (Neuro Probe, Gaithersburg, Maryland, USA) with an 8-µM pore membrane (Neuro Probe) in RPMI-1640 with 10% FBS as the chemoattractant in the lower chamber. Briefly, AGS cells were added to the upper chamber with nicotine for 24 h, the non-invading cells on the upper surface of each membrane were removed from the chamber by using cotton swabs, and the invading cells on the lower surface of each membrane were stained using the Quick-Diff stain kit (Becton-Dickinson, Franklin Lakes, NJ, USA). After two washes with water, the chambers were allowed to air dry. The number of invading cells was counted using a phase-contrast microscope. 

### 5.9. Statistics Analysis

Quantitative data were analyzed using a one-way analysis of variance followed by Tukey’s honestly significant difference tests between individual groups. Data are expressed as the mean ± SEM. A value of *p* < 0.05 was considered to be significant. The statistical software package Prism 5.0 (GraphPad Software, La Jolla, CA, USA) was used for analysis.

## Data Availability

The data presented in this study are available in the article.
